# Correction to “Diffuse large B‐cell lymphoma in large intestine presenting as multiple polypoid lesions”

**DOI:** 10.1002/jgh3.13096

**Published:** 2024-05-27

Yang R, Lan T, Tong H. **Diffuse large B‐cell lymphoma in large intestine presenting as multiple polypoid lesions**. *JGH Open*. 2023;**7**:520–521.

The authors' affiliation “*West China School of Medicine, †Department of Gastroenterology and Hepatology, West China Hospital and ‡Lab of Gastroenterology and Hepatology, West China Hospital, Sichuan University, Chengdu, China” was incorrect. This should have read: “Department of Gastroenterology and Hepatology, West China Hospital, Sichuan University, Chengdu, China.”

We apologize for this error.

